# Treatment of transfusion requiring anemia in a Quick Diagnostic Unit integrated in an Emergency Department Setting

**DOI:** 10.1186/1757-7241-21-S2-A48

**Published:** 2013-09-09

**Authors:** Charlotte Stenqvist, Søren Wistisen Rasmussen, Thomas Andersen Schmidt

**Affiliations:** 1 The Emergency Department, Holbaek University Hospital, Denmark

## Background

The establishment of a Quick Diagnostic Unit (QDU) in an Emergency Department (ED) setting has allowed expeditious blood transfusion of anaemic patients. The purpose of the study was to establish the mode of referral, describe the clientele, determine the underlying diseases and the Hb level of the referred patients.

## Methods

Chart review of an 8 month period. Values were given as mean ± SEM. Significance was evaluated using Student’s two-tailed t-test for unpaired observations. The level of significance was p < 0.05.

## Results

We found 108 patients. 71% was referred to hospital by their general practitioner and 18% of the patients came from oncological departments. In the given period we treated around 4 patients each week. 25 patients were admitted more than once, on average they came every 42nd day. Two thirds of the patients only stayed for a few hours.

55 patients had a diagnosed cancer, 29 were men and 26 were women. 53 patients had a nonmalignant disease, 26 were men and 27 were women. The mean age for oncological patients was 73.8 ± 1.3 (n = 55) years and for nonmalignant patients 75 ± 1.8 years (n = 53) (p > 0.6).

Oncological patients were given SAG-M transfusions at a Hb level of 5.0 ± 0.09 mMol/L (80.4 ± 1.4 g/L). Nonmalignant patients received SAG-M at a Hb level of 4.7 ± 0.07 mMol/L (75.7 ± 1.1 g/L) (p <0.05).

On average patients with malignant disease tended to receive less blod than patients with nonmalignant diseases (p=0.06), i.e. 2.2 ± 0.1 vs. 2.5 ± 0.1 SAG-M per contact. This however in clinical practice amounts to 2 SAG-M for both patient categories.

## Conclusion

SAG-M transfusion may be given expeditiously in a QDU setting to elderly patients. On average oncological patients received SAG-M at a higher Hb level than other anemic patients. The transfusion tigger for patients with nonmalignant disease appears to comply with national guidelines.

